# Impact of the COVID-19 pandemic on the symptomatology and routine of medicated patients with obsessive-compulsive disorder

**DOI:** 10.47626/1516-4446-2023-3333

**Published:** 2024-03-25

**Authors:** Natália B. Almeida, Maria Paula Maziero, Tais Tanamatis, Danel Lucas da Conceição Costa, Roseli G. Shavitt, Marcelo Q. Hoexter, Marcelo C. Batistuzzo

**Affiliations:** 1 Departamento de Psiquiatria, Faculdade de Medicina, Universidade de São Paulo, São Paulo, SP, Brazil; 2 Centro Universitário São Camilo, São Paulo, SP, Brazil; 3 Department of Psychiatry & Behavioral Sciences, University of Texas Health Science Center at Houston, Houston, TX, USA; 4 Departamento de Métodos e Técnicas, Pontifícia Universidade Católica de São Paulo, São Paulo, SP, Brazil

**Keywords:** COVID-19, OCD, suicide, symptomatology

## Abstract

**Objectives:**

To study the impact of coronavirus disease 2019 on the routine of patients with obsessive-compulsive disorder (OCD) and changes in symptoms and suicidal-related behavior, mainly in those with cleaning symptoms.

**Methods:**

In this cross-sectional study, 58 patients completed an online self-report questionnaire that included the Obsessive-Compulsive Inventory-Revised, Coronavirus Stress and Traumatic Events Scale, Coronavirus Health Impact Survey, Beck Anxiety and Beck Depression inventories, and Suicide-Related Behaviors Questionnaire. Comparisons were made with another pre-pandemic sample (n=524) regarding the last three measures.

**Results:**

During the pandemic, the patients spent more days inside their homes (χ^2^ = 33.39, p = 0.007), changed their alcohol consumption patterns (χ^2^ = 87.6, p < 0.001), and increased social media usage (χ^2^ = 68.83, p < 0.001). Participants with cleaning symptoms did not significantly differ from the others in relation to stress, anxiety/depressive symptoms, or suicidal-related behaviors. Finally, our sample did not differ from an equivalent OCD sample assessed before the pandemic in terms of anxiety and depressive symptom severity or suicidal-related behaviors.

**Conclusion:**

Overall, patients with OCD showed no lifestyle changes associated with higher stress levels during the pandemic. Patients with and without cleaning symptoms and patients before and during the pandemic presented similar results.

## Introduction

Obsessive-compulsive disorder (OCD) affects approximately 1-3% of the population.^1^ Patients with OCD who experience stressful events have a 42% higher probability of a chronic course, which commonly leads to increased negative events throughout life, such as adverse health changes and traumatic events.^2^ Greater symptom severity is also correlated with higher numbers of these events.^3^ Various studies have attempted to investigate the impact of stressful events on individuals with OCD, particularly regarding the coronavirus disease 2019 (COVID-19) pandemic. However, the findings are inconsistent: while one study^4^ showed stable trajectories of OCD symptoms over time, a systematic review^5^ of 59 studies with different demographic groups indicated that OCD symptoms worsened after the pandemic.^5^

Therefore, the present study investigated the impact of the COVID-19 pandemic on a sample of Brazilian patients with OCD who were being treated when invited to participate. Given that the pandemic involved viral contamination risk, there was a basic need for greater hygiene, which may have led to higher stress levels for patients with cleaning symptoms. Our specific aims were: 1) to analyze lifestyle and emotional changes and stress level, determining whether patients with cleaning symptoms experienced more changes in their daily routines than other patients, and 2) to evaluate anxiety and depressive symptoms and suicidal-related behaviors during the pandemic compared to a patient sample assessed before the pandemic.

We hypothesized that the pandemic would increase the presence of stressful events in individuals with OCD, especially in patients with cleaning symptoms, due to the need for greater attention to hygiene. Specifically, we expected that patients would experience: 1) higher stress levels and lifestyle changes, and 2) that anxiety and depressive symptoms and suicide-related behaviors would increase.

## Methods

The sample consisted of 58 patients with a primary DSM-5 diagnosis of OCD who were undergoing treatment at the Instituto de Psiquiatria, Hospital das Clínicas, Faculdade de Medicina Universidade de São Paulo. Exclusion criteria were age under 18 years, intellectual disability, and significant neurological comorbidities.

In this cross-sectional study, the participants answered self-report scales between May and September 2021. The online assessment consisted of the following scales: Obsessive-Compulsive Inventory-Revised (OCI-R), which investigates the presence and severity of obsessions and compulsions^6^; the Beck Anxiety (BAI) and Beck Depression (BDI) inventories^7^,^8^; the Coronavirus Stress and Traumatic Events Scale (COROTRAS), which explores lifestyle changes associated with the pandemic and the emotions (fear, helplessness, disgust, anger, guilt, shame, and sadness) related to the main lifestyle change^9^; the Coronavirus Health Impact Survey (CRISIS),^10^ which assesses life changes and stress by asking about the respondent’s lifestyle before and during the pandemic. Since a recent meta-analysis suggested that individuals with OCD have more suicidal ideation than the general population,^11^ participants were also asked to respond to a questionnaire on suicide planning and attempts by the Consórcio Brasileiro de Pesquisa em Transtornos do Espectro Obsessivo-Compulsivo (C-TOC).^12^ Hypothesizing that suicide-related behaviors would increase among patients with OCD during the pandemic, we used the C-TOC database (data collected before the pandemic) for comparison with the present sample.^12^ This database comprises 524 individuals with OCD who lived in the state of São Paulo and were evaluated between 2005 and 2007.

JASP version 0.16.2 was used for the statistical analyses. The chi-square test was performed to compare categorical variables before and during the pandemic (CRISIS data) and the suicide variables across samples. Kendall correlations were calculated between COROTRAS, OCI-R, BAI, and BDI data to determine whether greater symptom severity was associated with greater lifestyle changes. The same analyses were performed between the CRISIS and OCI-R, BAI, and BDI data to determine the association with a higher frequency of stressful events. The Mann-Whitney test was used for between-group comparisons of patients with and without cleaning symptoms (or before and during pandemics), since the majority of the COROTRAS variables did not pass the normality and equality of variance assumptions.

### Ethics statement

This study was approved by the institutional ethics committee (CAAE 45614521.4.0000.0068), and all participants provided written informed consent.

## Results

### Demographics

The mean participant age was 41.4 years (SD = 12.7); 55% of the participants were women (χ^2^ = 0.311, p = 0.577), and 76% were White (χ^2^ = 23.4; p < 0.001). The majority lived in the city of São Paulo. Approximately 20% of the sample reported being diagnosed with COVID-19 (17.2% tested positive and 3.4% were diagnosed medical without a test), and 75% received at least one dose of the COVID-19 vaccine, with approximately 50% completing the two-dose regimen. The patients were already undergoing either medical (96%) or psychological (31%) treatment for OCD, and 58% reported no difficulties in continuing their treatment through remote assistance. Of the 524 patients in the C-TOC database, 51.9% were women; their mean age was 35.0 (SD = 11.8) years, and 87.5% were White.

### Clinical assessment

**Figure 1:**
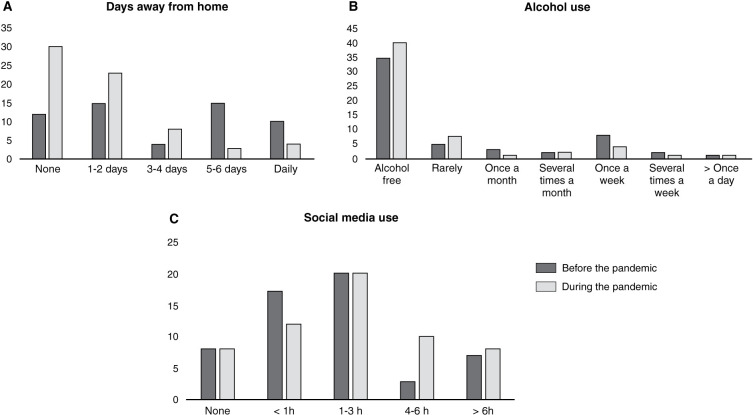
Coronavirus Health Impact Survey (CRISIS) results comparing patient lifestyles during and 3 months before the pandemic: A) Days participants stayed away from home (p = 0.007). B) Alcohol use (p < 0.001). C) Social media use (p < 0.001).

The CRISIS data indicated that the participants stayed more days at home (χ^2^ = 33.4, p = 0.007) and that their digital media usage time increased (χ^2^ = 68.8 and p < 0.001). Alcohol intake also decreased (χ^2^ = 87.6 and p < 0.001) compared to 3 months before the pandemic (Figure 1). The frequency distributions for specific CRISIS questions are shown in the supplementary material (Table S1, available online-only). The OCIclean+ and OCIclean- groups did not differ in COROTRAS scores (all p > 0.10) or in anxiety or depression symptoms (p = 0.341 and 0.941, respectively), although the OCIclean+ group was more worried about friends and family getting infected (CRISIS item 25; Table S2, available as online-only supplementary material).

No significant results were found in specific CRISIS questions related to stress exposure and previous contact with COVID-infected individuals (Table S3, available as online-only supplementary material). There were also no differences between our sample and the C-TOC sample regarding anxiety and depressive symptoms and suicide-related behaviors (Table 1). In our sample, total OCI-R scores did not differ between patients with and without suicidal-related behaviors. Finally, there were no correlations between COROTRAS, OCI-R, BAI, and BDI (all p > 0.05) scores, although specific CRISIS items (26, 41, and 27) were positively correlated with OCI-R, BAI, and BDI, respectively (Table S4, available online only). There were no significant correlations for these variables in the OCIclean+ sample (Table S5, available as online-only supplementary material).

## Discussion

In contrast to our initial hypotheses, the results indicated that patients with OCD generally showed no lifestyle changes due to higher stress levels during the pandemic. Furthermore, patients with cleaning symptoms did not report more severe stress than those without them, and there was no difference in suicide-related behaviors compared to a pre-pandemic sample. These results differ from previous studies that found a worsening of obsessive-compulsive symptoms and quality of life during the pandemic.^5^ However, similar to our findings, other national^13^ and international^14^ studies found no major changes in psychiatric symptoms, such as OCD, after the pandemic began. It may be that cultural differences or specificities from this sample (such as being in treatment) could have influenced these outcomes.

Several predictors of COVID-19-related morbidity and mortality have been identified: demographic-geographic factors (male-to-female ratio, population density, urbanization, education, temperature, and religious diversity), political-legal factors (democracy, political corruption, female leadership, legal system strength, and public trust in government), and sociodemographic factors (income inequality, life satisfaction, and technological advancement). Considering that the development or worsening of psychiatric symptoms may be related to the impact of the pandemic in terms of morbidity and mortality in a specific location, these factors may also explain the divergent results of the present study.^4^

The fact that our sample was being treated for OCD at the time of assessment might explain why our initial hypotheses, which were based on the literature, were not confirmed. This result agrees with a recent review of 134 articles evaluating the impact of COVID-19 on several psychiatric symptoms, which found no changes in general mental health or anxiety symptoms, with only minimal worsening of depression symptoms.^15^ In contrast, other studies have found that the COVID-19 pandemic was associated with less effective exposure-response prevention therapy in most patients during the first several months of the pandemic: clinicians estimated that symptoms worsened in 38% of their patients during the pandemic and remained unchanged in 47% despite therapy.^16^ Exposure-response prevention therapy was less effective in those who endured financial distress or were medically at-risk for severe COVID-19, and it was less effective in adults than youth during the pandemic.^16^

It was also observed that patients spent less time away from home, most likely due to social isolation measures. As a result, they tended to use more social media. Interestingly, alcohol consumption decreased, which contradicts previous evidence of a significant increase in alcohol consumption during the pandemic.^17^ One possible explanation for this discrepancy is the online psychiatric treatment that most patients were receiving during the evaluations, which may have resulted in better understanding of their OCD and better coping skills for stressful situations. There were also no correlations between OCD symptoms and life changes during the pandemic, which could indicate an independent relationship between the number and diversity of OCD symptoms and emotions related to lifestyle change.

Of note, one of the main hypotheses of this study was not confirmed: although OCIclean+ and OCIclean- patients differed regarding “worries about friends and family getting infected” (CRISIS item 25), they were similar in all other items, indicating that the presence of contamination and washing symptoms did not significantly affect pandemic outcomes. Furthermore, our analyses revealed no significant increase in stress or psychiatric symptoms (anxiety or depression) in patients with cleaning symptoms, despite the changes the pandemic made to the daily routine, such as increased hand washing and social isolation.^11^ Taken together, our findings indicate that the pandemic did not particularly affect patients with this symptom dimension.

Since a meta-analysis suggested that individuals with OCD are at a considerably higher risk of suicide attempts and have more suicidal ideation than the general population,^11^ it was hypothesized that suicide-related behaviors would increase among patients during the pandemic. However, based on our findings, we cannot speculate whether the pandemic led to increased mental suffering, since we found no discrepancy in suicide-related behaviors compared to a pre-pandemic sample or increased anxiety and depressive symptoms. It should be pointed out that, unlike the meta-analysis, our study did not analyze healthy individuals, making it difficult to speculate about the frequency of suicidal-related behaviors of this population. In general, there is evidence that suicidal ideation increased with strict COVID-19 lockdowns.^18^ However, our study design did not allow us to detect variations in suicidal-related behaviors. Moreover, the fact that we did not assess lockdown conditions (only the number of days away from home) is a study limitation.

In conclusion, we found no increase in stress levels during the pandemic in this sample of OCD patients. No significant differences in stress levels could be attributed to cleaning symptoms. The results also indicated relative stability in participant lifestyle, with no discernible variations in the severity of depressive and anxiety symptoms or suicidal-related behaviors compared to a pre-pandemic sample. However, caution is required when interpreting these findings, considering that the sample size was relatively small and consisted of individuals receiving treatment. Moreover, the lack of pre-pandemic evaluations for these individuals adds a potential confounding factor; a longitudinal study would have been ideal to accurately ascertain how patient symptoms evolved during the pandemic. Finally, we collected no information about contact with symptom-eliciting materials, since we did not apply scales that assess avoidance behaviors.

## Disclosure

RGS has received speaker honoraria from Lundbeck. The other authors report no conflicts of interest.

## Figures and Tables

**Table 1:** Between-group comparison for suicide-related questions and Beck Anxiety and Depression scores

Question	C-TOC (n=524)	Current study (n=58)	χ^2^/U	p-value
Have you ever thought that life wasn’t worth living?	303 (62.3)	25 (49.0)	3.44	0.063†
Did you ever wish you were dead?	231 (47.6)	23 (45.1)	0.11	0.731†
Have you ever thought of committing suicide?	183 (37.7)	16 (31.4)	0.80	0.371†
Have you ever planned how to do it?	101 (20.1)	11 (21.5)	0.01	0.901†
Have you ever attempted suicide?	62 (12.7)	6 (11.7)	0.25	0.880†
Currently, do you think about committing suicide?	62 (12.7)	8 (16.0)	0.41	0.521†
Has anyone in your family ever tried to commit suicide?	87 (17.9)	8 (16.0)	0.12	0.728†
Has anyone in your family committed suicide?	78 (16.1)	10 (20.0)	0.49	0.481†
BAI (M [SD])	17.1 (11.9)	17.7 (10.9)	0.520	0.703‡
BDI (M [SD])	18.2 (11.4)	17.0 (9.6)	0.452	0.487‡

Data presented as n (%), unless otherwise specified.

BAI = Beck Anxiety Inventor; BDI = Beck Depression Inventor; C-TOC = Consórcio Brasileiro de Pesquisa em Transtornos do Espectro Obsessivo-Compulsivo; χ^2^ = chi-square test.

† χ^2^.

‡ Mann-Whitney test.

## References

[1] Mathes BM, Morabito DM, Schmidt NB (2019). Epidemiological and clinical gender differences in OCD. Curr Psychiatry Rep.

[2] van Oudheusden LJB, Eikelenboom M, van Megen HJGM, Visser HAD, Schruers K, Hendriks GJ (2018). Chronic obsessive-compulsive disorder: prognostic factors. Psychol Med.

[3] Adams TG, Kelmendi B, Brake CA, Gruner P, Badour CL, Pittenger C (2018 Jan-Dec:2). The role of stress in the pathogenesis and maintenance of obsessive-compulsive disorder. Chronic Stress (Thousand Oaks).

[4] Hezel DM, Rapp AM, Wheaton MG, Kayser RR, Rose SV, Messner GR (2022). Resilience predicts positive mental health outcomes during the COVID-19 pandemic in New Yorkers with and without obsessive-compulsive disorder. J Psychiatr Res.

[5] Linde ES, Varga TV, Clotworthy A (2022). Obsessive-compulsive disorder during the COVID-19 pandemic: a systematic review. Front Psychiatry.

[6] Foa EB, Huppert JD, Leiberg S, Langner R, Kichic R, Hajcak G (2002). The Obsessive-compulsive inventory: development and validation of a short version. Psychol Assess.

[7] Beck AT, Steer RA (1993). Beck Depression inventory: manual.

[8] Beck AT, Ward CH, Mendelson M, Mock J, Erbaugh J (1961). An inventory for measuring depression. Arch Gen Psychiatry.

[9] Fontenelle L, Muhlbauer JE, Albertella L, Eppingstall J (2020). The impact of coronavirus on individuals with problematic hoarding behaviours.

[10] Nikolaidis A, Paksarian D, Alexander L, Derosa J, Dunn J, Nielson DM (2021). The Coronavirus Health and Impact Survey (CRISIS) reveals reproducible correlates of pandemic-related mood states across the Atlantic. Sci Rep.

[11] Dell’Osso B, Benatti B, Arici C, Palazzo C, Altamura AC, Hollander E (2018). Prevalence of suicide attempt and clinical characteristics of suicide attempters with obsessive-compulsive disorder: a report from the International College of Obsessive-Compulsive Spectrum Disorders (ICOCS). CNS Spectr.

[12] Miguel EC, Ferrão YA, do Rosário MC, Mathis MA, Torres AR, Fontenelle LF (2008). The Brazilian Research Consortium on obsessive compulsive spectrum disorders: recruitment, assessment instruments, methods for the development of multicenter collaborative studies and preliminary results. Braz J Psychiatry.

[13] Brunoni AR, Suen PJC, Bacchi PS, Razza LB, Klein I, Dos Santos LA (2023). Prevalence and risk factors of psychiatric symptoms and diagnoses before and during the COVID-19 pandemic: findings from the ELSA-Brasil COVID-19 mental health cohort. Psychol Med.

[14] Chang D, Chang X, He Y, Tan KJK (2022). The determinants of COVID-19 morbidity and mortality across countries. Sci Rep.

[15] Sun Y, Wu Y, Fan S, Dal Santo T, Li L, Jiang X (2023). Comparison of mental health symptoms before and during the covid-19 pandemic: evidence from a systematic review and meta-analysis of 134 cohorts. BMJ.

[16] Storch EA, Sheu JC, Guzick AG, Schneider SC, Cepeda SL, Rombado BR (2021 Jan). Impact of the COVID-19 pandemic on exposure and response prevention outcomes in adults and youth with obsessive-compulsive disorder. Psychiatry Res.

[17] Organização Pan-Americana de Saúde (OPAS) (8 de setembro de 2020). Uso de álcool durante a pandemia de COVID-19 na América Latina e no Caribe.

[18] Killgore WDS, Cloonan SA, Taylor EC, Allbright MC, Dailey NS (2020). Trends in suicidal ideation over the first three months of COVID-19 lockdowns. Psychiatry Res.

