# Supplementary material for: Impact of the COVID-19 pandemic on the symptomatology and routine of medicated patients with obsessive-compulsive disorder
Source: Braz J Psychiatry. 2024 Mar 25;46:e20233333. doi: 10.47626/1516-4446-2023-3333 (PMC11189129; doi:10.47626/1516-4446-2023-3333)
Supplement: Supplementary file 1 [file bjp-46-e20233333-s001.pdf]

http://doi.org/10.47626/1516-4446-2023-3333

Table S1 Distribution of CRISIS item responses related to exposure to stressful events in the entire sample (n = 58)

Question	Not at all	Slightly	Moderately	Very	Extremely
25 - How worried have you been about your friends and family getting infected?	8 (13.7%)	16 (27.5%)	9 (15.5%)	17 (29.3%)	8 (13.7%)
26 - How worried have you been about your physical health being influenced by COVID-19?	12 (20.6%)	14 (24.1%)	9 (15.5%)	14 (24.1%)	9 (15.5%)
27 - How worried have you been about your mental health being influenced by COVID-19?	9 (15.5%)	10 (17.2%)	11 (18.9%)	17 (29.3%)	11 (18.9%)
28 - How much are you reading or talking about Coronavirus/COVID-19?	0 (0%)	6 (10.3%)	19 (32.7%)	27 (46.5%)	6 (10.3%)
29 - Has the Coronavirus/COVID-19 crisis in your area led to any positive changes in your life?	0 (0%)	25 (43.1%)	14 (24.1%)	19 (32.7%)	0 (0%)
34 - How stressful have the restrictions on leaving home been for you?	7 (12%)	12 (20.6%)	13 (22.4%)	13 (22.4%)	13 (22.4%)
40 - How stressful have the restrictions on leaving home been for you?	13 (22.4%)	15 (25.8%)	15 (25.8%)	9 (15.5%)	6 (10.3%)
41 - How much has the cancellation of important events (such as graduation, prom, vacation, etc.) in your life been difficult for you?	16 (27.5%)	14(24.1%)	17(29.3%)	7 (12%)	4(6.8%)
45 - How much hope do you have about the COVID-19 crisis where you live will be resolved soon?	10 (17.2%)	18 (31.0%)	14 (24.1%)	12 (20.6%)	4 (4.8%)

http://doi.org/10.47626/1516-4446-2023-3333

Table S2 Comparison between patients with (n = 44) and without (n = 14) cleaning symptoms regarding CRISIS items specifically related to exposure to stressful events

	**W**	**p-value**†
crisis25	462.000	**0.004**
crisis26	421.000	0.037
crisis27	421.500	0.035
crisis28	355.000	0.363
crisis29	336.500	0.586
crisis34	338.500	0.577
crisis40	342.000	0.532
crisis41	296.500	0.836
crisis45	260.000	0.374

† Mann-Whitney U test, Bonferroni correction (< 0.0056).
Significant p-values in bold font.

Table S3 Comparison between patients with (n=18) and without (n=40) exposure to COVID-infected individuals regarding CRISIS items specifically related to exposure to stressful events

	**W**	**p-value**†
crisis25	364.000	0.952
crisis26	337.500	0.705
crisis27	369.000	0.884
crisis28	329.500	0.587
crisis29	346.500	0.815
crisis34	258.000	0.081
crisis40	239.500	0.038
crisis41	307.000	0.362
crisis45	248.000	0.054

† Mann-Whitney U test, Bonferroni correction (<0.0056).

http://doi.org/10.47626/1516-4446-2023-3333

Table S4 Correlation coefficients between CRISIS items specifically related to exposure to stressful events in the entire sample (n=58) and total OCI-R, BAI, and BDI scores.

	**OCI-R**	**BAI**	**BDI**
CRISIS 25	0.269	0.190	0.258
CRISIS 26	**0.300**†	0.245	0.272
CRISIS 27	0.230	0.239	**0.391**†
CRISIS 28	0.078	0.127	0.238
CRISIS 29	0.109	0.084	0.101
CRISIS 34	0.069	0.201	0.246
CRISIS 40	0.012	0.224	0.280
CRISIS 41	-0.057	**0.340**†	0.224
CRISIS 45	-0.207	0.051	-0.027

† Kendall's Tau Correlation, Bonferroni correction (separately for OCI-R, BAI, and BDI).
Significant p-values in bold font (alpha value < 0.0056).

Table S5 Correlation coefficients between CRISIS items specifically related to exposure to stressful events in OCD patients with cleaning symptoms (n=44) and total OCI-R, BAI, and BDI scores.

	**OCI-R**	**BAI**	**BDI**
CRISIS 25	0.297	0.228	0.301
CRISIS 26	0.243	0.204	0.126
CRISIS 27	0.230	0.169	0.267
CRISIS 28	0.027	0.112	0.194
CRISIS 29	-0.038	0.173	0.129
CRISIS 34	0.044	0.060	0.188
CRISIS 40	-0.008	0.101	0.269
CRISIS 41	-0.140	0.338	0.247
CRISIS 45	-0.174	-0.051	-0.083

† Kendall's Tau Correlation, Bonferroni correction (separately for OCI-R, BAI, and BDI).
Alpha value < 0.0056.
